# Enhanced Pyroelectric Response of Lithium Niobate Crystals for Infrared Detection Applications

**DOI:** 10.3390/s26041141

**Published:** 2026-02-10

**Authors:** Chencheng Zhao, Ziqi Liu, Qinglian Li, Jun Sun, Jingjun Xu

**Affiliations:** 1 School of Physics, Nankai University, Tianjin 300071, China; 1120210066@mail.nankai.edu.cn (C.Z.); 2120230242@mail.nankai.edu.cn (Z.L.); liql@nankai.edu.cn (Q.L.); 2 Chongqing Impartial Optoelectronic Co., Ltd., Chongqing 402760, China; 3 Research Center for Crystal Materials, Xinjiang Key Laboratory of Functional Crystal Materials, Xinjiang Technical Institute of Physics and Chemistry, Chinese Academy of Sciences, Urumqi 830011, China

**Keywords:** lithium niobate crystal, pyroelectric effect, pyroelectric infrared detection, response voltage

## Abstract

**Highlights:**

**What are the main findings?**

- The figures of merit of lithium niobate crystals were enhanced approximately four-fold at room temperature.
- The response voltage of a pyroelectric infrared detector was increased eight-fold, making it suitable for practical applications.

**What are the implications of the main findings?**

- A simple and efficient method for significantly enhancing the pyroelectric coefficient of lithium niobate crystals was devised.
- The method provides an environmentally friendly pyroelectric material with excellent performance.

**Abstract:**

This work addresses the low pyroelectric coefficient that limits the practical application of lithium niobate (LN) crystals. A defect modulation process based on reduction annealing treatment is proposed. This reduction annealing treatment increased the pyroelectric coefficient of LN crystals maximally to 3.362 × 10^−4^ C/m^2^K. At room temperature, the voltage responsivity figure of merit (F_V_) and detectivity figure of merit (F_D_) were both improved more than three-fold. All material properties exceeded those of commercial lead zirconate titanate (PZT) ceramic. This process achieves the simultaneous modulation of high pyroelectric coefficients and low impedance in LN crystals. Based on the LN crystals with optimized properties, pyroelectric infrared detectors (center wavelength 9.4 μm) without external matching resistors were prepared. The response voltage of the detector reached 2.8 times that of commercial PZT detectors while exhibiting lower noise, and has achieved practical applicability. This work provides a simple and efficient method for developing environmentally friendly, low-cost, high-sensitivity pyroelectric infrared detectors. It also establishes the foundations for the application of LN crystals in emerging pyroelectric detection fields.

## 1. Introduction

All objects in nature above absolute zero emit infrared radiation. Pyroelectric materials convert temperature changes caused by external infrared radiation into electrical signals through the pyroelectric effect. This physical mechanism forms the operational foundation of pyroelectric infrared detection (PIRD). PIRD offers advantages such as there being no cooling requirement, a fast response rate, a wide operating frequency range, ease of integration, and low fabrication costs. These attributes have enabled its wide application in civilian fields, such as human motion detection, gas analysis, non-contact temperature measurement, and infrared spectroscopy [1,2,3]. They also play a key role in military technologies, including infrared night vision and target tracking [4]. Recently, the development of PIRD technology has spurred research on several frontier directions. These include flexible wearable electronic devices [5], self-powered temperature sensing [6], thermal energy harvesting [7], pyroelectric catalytic degradation [8], and ultraviolet photodetection [9].

PIRD technology for human bodies has achieved large-scale application in healthcare, security monitoring, and smart home scenarios [10]. Lead zirconate titanate (PbZr_1−x_Ti_x_O_3_, PZT)-based ceramic materials are among the most widely used and technologically mature materials in commercial detectors. They offer a high pyroelectric coefficient (2 × 10^−4^~4 × 10^−4^ C/m^2^K), a relatively high Curie temperature (473~573 K), and require no bias electric field [11]. However, the extremely high dielectric constant of PZT (typically 1000~3000) severely limits device response speed [12,13]. Furthermore, PZT-based ceramics contain 60~70% lead, which poses a significant environmental risk. Consequently, extensive research has been conducted on lead-free ferroelectric materials [14,15,16]. Another commercially mature material is single-crystal lithium tantalate (LiTaO_3_, LT). It has a pyroelectric coefficient of approximately 1.9 × 10^−4^ C/m^2^K, a high Curie temperature of 938 K, a relatively low dielectric constant (~40), excellent temperature stability, and can be grown as large single crystals via the Czochralski method [17,18]. However, the extremely high resistivity of LT crystals requires the integration of high-precision, high-resistance load resistors inside the detector to achieve impedance matching with the preamplifier. This increases fabrication cost and conflicts with the technical approach for reducing detection noise.

Lithium niobate (LiNbO_3_, LN) crystals have a similar structure to LT crystals, while exhibiting a higher Curie temperature (~1415 K), a lower melting point, and lower preparation costs, but the pyroelectric coefficient is only about 0.8 × 10^−4^ C/m^2^K. Consequently, the application of their pyroelectric effect has long been overlooked. Congruent lithium niobate crystals are rich in intrinsic defects due to lithium deficiency, which allows for many of their properties to be significantly tuned through compositional control, valence state modulation, and doping engineering [19]. However, current research on optimizing pyroelectric performance is limited. Some researchers have reported enhancements in the pyroelectric properties of doped LN crystals. Through doping, the pyroelectric coefficient of Fe: LN crystals increased to 2.3 × 10^−4^ C/m^2^K at 323 K [20]; 2 mol% Zr: LN crystals showed significant improvement in voltage responsivity figure of merit and detectivity at 323 K [21]; and 0.2 mol% Cr: LN crystals achieved the pyroelectric coefficient of 2.3 × 10^−4^ C/m^2^K at room temperature [22]. Additionally, Jinan University used laser surface treatment technology to achieve valence state modulation of LN crystals, which increased the pyroelectric voltage of LN crystals 1.3-fold [23]. Nevertheless, the current modulation methods and processes are relatively complex, and performance consistency is difficult to guarantee. This still limits the practical application of LN in pyroelectric infrared detection. Therefore, if a simple and efficient method can be developed to simultaneously enhance the pyroelectric coefficient and reduce the resistivity of LN crystals, LN crystals will hold great promise for replacing PZT ceramic materials. Being a lead-free and environmentally friendly pyroelectric material, LN crystals could find widespread application in infrared detection.

This work addresses the low pyroelectric coefficient of LN crystals that limits their practical application, reduction annealing treatment technology is used to modulate the pyroelectric properties. By comparing the thermal properties and electrical properties of LN crystals with different reduction annealing states, and combined with the regulatory mechanism of the reduction annealing process, the regulation patterns of the pyroelectric properties in LN crystals were analyzed. Based on these, pyroelectric infrared detection devices were prepared using the performance-optimized LN crystals, and their detection performance was characterized and analyzed. Furthermore, the results were also compared with commercial PZT ceramic pyroelectric detectors (D203S).

## 2. Materials and Methods

### 2.1. Sample Preparation

#### 2.1.1. Crystal Preparation

Polycrystalline powders for crystal growth were prepared from high-purity (99.99%) raw Li_2_CO_3_ and Nb_2_O_5_ powders with a composition of Li/Nb = 48.6/51.4 (molar ratio) by weighing, mixing, reacting, and melting. In an air atmosphere, a 4-inch *z*-axis CLN crystal was prepared by the Czochralski method, followed by annealing and single-domain treatment. The wafers were directionally cut into z-cut wafers with dimensions of *x* × *y* × *z* = 25 mm × 25 mm × 0.5 mm. LN crystals were subjected to reduction annealing treatment in a tube furnace with controllable atmosphere. LN wafers were placed in a quartz tube, which was evacuated to a vacuum level of (9 ± 1) Pa. The temperature was increased to 653 K at a heating rate of 10 K/min. The reduction annealing times were 0 min, 10 min, 20 min, 30 min, 40 min, and 50 min. The resulting LN wafer samples were designated LN-1#, LN-2#, LN-3#, LN-4#, LN-5#, and LN-6#. The LN-1# sample was cooled directly to room temperature after reaching the reduction annealing temperature, without an isothermal holding stage. The LN crystal without reduction annealing treatment was designated as RAW-LN.

On one surface of the LN wafer samples, sixteen points were taken at equal intervals in a 4 × 4 grid. Φ3 mm circular electrodes were printed using conductive silver paste. The area of a single electrode was 7.065 mm^2^. The opposite surface had a 24 mm × 24 mm square electrode. These served as pyroelectric coefficient test samples. The reducing annealing LN wafers were directionally cut into *x* × *y* × *z* = 10 mm × 10 mm × 0.5 mm. Wafers with graphite spray on both surfaces were used as thermal properties test samples. Wafers with printed silver electrodes on both surfaces were used as electrical properties test samples.

#### 2.1.2. Detector Preparation

**Figure 1:**
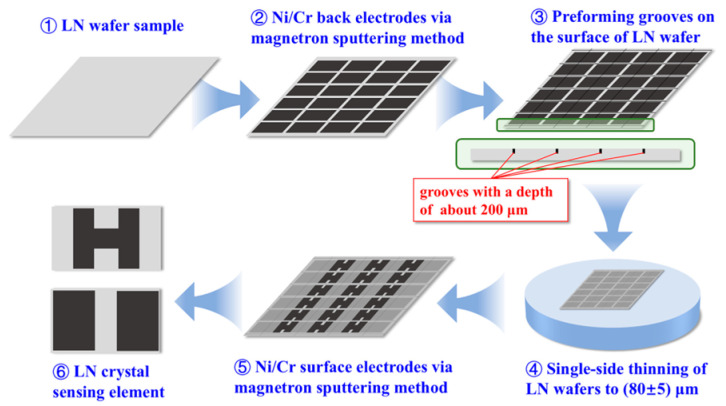
The process flow for the sensing elements of pyroelectric infrared detectors.

The pyroelectric material sensing elements are the core component of PIRDs. The process flow of the sensing element is shown in Figure 1. A 100 nm thick Ni/Cr metal electrode was deposited on one surface of the reduced LN wafer via the magnetron sputtering method. This side electrode is the back electrode of the sensing element. Grooves with a depth of about 200 μm were pre-formed on this surface. The other surface was then ground by mechanical grinding to reduce the wafer thickness to (80 ± 5) μm. The pre-formed grooves divided the wafer into sensing elements measuring 3 mm × 5 mm. Finally, electrodes of the same material and thickness were deposited on the other surface via magnetron sputtering. This is the front electrode of the sensing element. LN crystal sensing elements for PIRD were obtained.

To enable performance comparison with the commercial PZT ceramic pyroelectric detectors, the PIRDs of LN crystals prepared in this work were consistent with the commercial PZT ceramic detector in terms of internal structure, electronic components, packaging method, and pin definition. The PIRDs of LN crystals in this work operate in voltage mode, with the LN crystal sensing element and junction field-effect transistor (JFET) using TO-5 packaging. The LN crystal sensing elements were connected in series for temperature compensation. The JFET provides impedance matching and signal amplification. A narrowband infrared filter with a transmission wavelength of 7 μm to 14 μm is encapsulated at the window. It transmitted infrared radiation only at a central wavelength of 9.4 μm, filtering out ambient light and improving detection accuracy.

In this work, the commercial PZT ceramic pyroelectric detectors (TELESKY, Shenzhen, China) were off-the-shelf products with the model number D203S; the detector sample numbers were PZT-1# and PZT-2#.

### 2.2. Characterization

#### 2.2.1. Properties of Crystals

The pyroelectric coefficient (*p*) of LN crystals was measured by the dynamic charge integration method, and the temperature variation range was ±1.5 K. The thermal diffusivity (*α_z_*), thermal conductivity (*k_z_*), and mass specific heat capacity (*C_p_*) along the z-axis direction of the LN crystal samples were measured using a laser thermal conductivity analyzer (LFA467, NETZSCH, Selb, Germany). The resistivity (*ρ*) of LN crystals was measured using a high-resistance meter (TH2690, Tonghui, Changzhou, China) based on the voltage–current method. The dielectric constant (*ε_r_*) and dielectric loss (tan*δ*) of the LN crystals were measured by a precision impedance analyzer (E4990A, Keysight, Santa Rosa, CA, USA). The piezoelectric strain constant (*d*_33_) of the LN crystal samples was measured using the quasi-static method. Thermal properties were measured over a temperature range of 298~423 K. All other measurements were conducted at room temperature.

#### 2.2.2. Detection Performance

**Figure 2:**
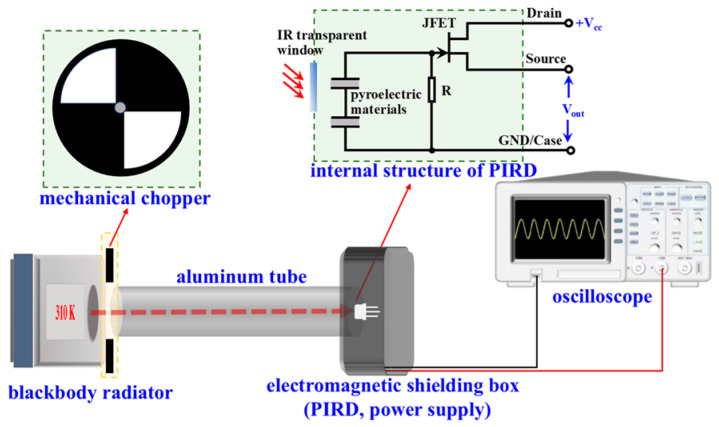
The pyroelectric response signal test system for pyroelectric infrared detectors.

The PIRD was measured at an environmental temperature of 300 K. Figure 2 shows the schematic of our self-built pyroelectric response test system. The blackbody radiation source (DY-HTY, D-MEI Instrument, Tai’an, China) emitted infrared radiation signals, and its temperature was set at 310 K to simulate human body temperature. This temperature corresponded to an infrared radiation central wavelength of 9.4 μm, which was consistent with the central wavelength of the narrowband filter in the sensor. A mechanical chopper modulated the infrared radiation signal at different frequencies. The detector and a 3.7 V power supply battery were enclosed in an aluminum shielding box, which was grounded. The distance between the infrared radiation source and the detector was 400 mm. An oscilloscope (MSO8104, Rigol, Suzhou, China) amplified the detector output signal by 10× and provided real-time monitoring.

## 3. Results and Discussion

### 3.1. Effects of Reduction Annealing Treatment on the Properties of Lithium Niobate Crystals

Since LN crystals were grown in an air atmosphere, oxygen annealing treatment cannot readily modify the composition or lattice defect structure of LN crystals. Although higher oxygen content or higher pressure may yield better results, the practical value is limited. Li-rich atmosphere annealing treatment changes LN crystal composition through Li ion diffusion; however, the pyroelectric coefficient does not show significant improvement with different Li contents [24]. In contrast, reduction annealing treatment enables lattice deoxygenation, with a degree of oxygen loss that can be controlled by adjusting the reduction annealing conditions. This provides a simple, rapid, and controllable modulation approach that is more efficient and cost-effective in practice. Therefore, reduction annealing treatment was primarily considered in this work to modulate the pyroelectric properties of LN crystals.

**Figure 3:**
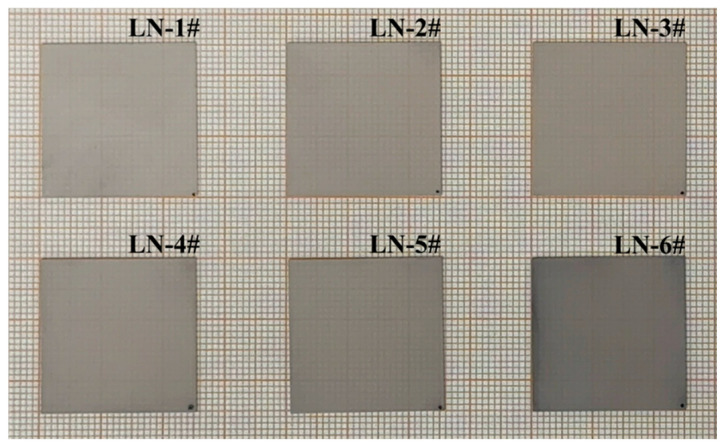
LN wafer samples prepared by different reduction annealing processes.

During the reduction annealing treatment, LN wafer samples were in an oxygen-deficient environment, causing two oxygen atoms in the lattice combined into oxygen molecules and escape to the gas phase. Oxygen vacancies and two free electrons remained in the lattice. These free electrons were captured by anti-site Nb (${\mathrm{Nb}}_{\mathrm{Li}}^{4+}$) or electron pairs (${\mathrm{Nb}}_{\mathrm{Li}}^{4+}-{\mathrm{Nb}}_{\mathrm{Nb}}^{5+}$) to form small polarons (${\mathrm{Nb}}_{\mathrm{Li}}^{3+}$) and bipolarons (${\mathrm{Nb}}_{\mathrm{Li}}^{3+}-{\mathrm{Nb}}_{\mathrm{Nb}}^{4+}$). These defects caused coloration of LN crystals after reduction annealing [25,26,27]. As the reduction annealing time increased, the degree of reduction annealing intensified, and defect concentration increased. LN wafer samples showed different colors from light gray to dark gray, as shown in Figure 3.

The pyroelectric coefficient, thermal properties, and electrical properties measured for LN crystals at different reduction annealing processes are shown in Table 1. After reduction annealing treatment, the pyroelectric coefficient of LN crystals first increases and then decreases with increasing reduction annealing time. The pyroelectric coefficient of LN-2# increased by more than three times compared to RAW-LN, which is comparable to that of commercial PZT ceramic materials (~3.4 × 10^−4^ C/m^2^K). The resistivity of LN crystals gradually decreases with increasing reduction annealing time. Additionally, other thermal properties and electrical properties did not change significantly during short reduction annealing treatment.

Congruent lithium niobate (CLN) crystals contain lithium deficiency, which creates lithium vacancies $V_{\mathrm{Li}}^{-}$ and ${\mathrm{Nb}}_{\mathrm{Li}}^{4+}$ intrinsic defects in the lattice. Oxygen atoms occupying the $V_{\mathrm{Li}}^{-}$-O octahedra and ${\mathrm{Nb}}_{\mathrm{Li}}^{4+}$-O octahedra exhibit lower thermodynamic stability than those at normal lattice sites, including localized lattice distortion. These oxygen atoms preferentially escape during the reduction annealing processes, forming oxygen vacancies $V_{O}$. Under the combined effect of these defects and intrinsic defects, the relative motion of Nb atoms at normal sites and oxygen octahedra along the *z*-axis is affected, leading to an increase in the pyroelectric coefficient of LN crystals (e.g., LN-1# and LN-2#). With the deepening of reduction annealing, oxygen atoms at other sites, especially normal lattice sites, begin to escape, and the pyroelectric coefficient of LN crystals then start to decrease, as exemplified by LN-3#. With more oxygen atoms escaping from these sites, the pyroelectric coefficient of LN crystals became even lower than that of RAW-LN crystals, such as LN-6#.

The decrease in LN crystal resistivity stems from the increased concentration of free electrons introduced by reduction annealing. A suitable increase in free electrons facilitates heat absorption, leading to a more rapid temperature response and thus improves the pyroelectric properties of LN crystals [28]. However, as free electrons increased, the induced charges generated by spontaneous polarization changes induced by external temperature variations are rapidly “neutralized”, leading to a decrease in the experimentally measured pyroelectric coefficient. Through the reduction annealing process optimization, it is possible to achieve synergistic control over both enhancing the pyroelectric coefficient and reducing the impedance of LN crystals.

The other thermal properties and electrical properties of LN crystals did not change significantly after reduction annealing. This means the enhancement of pyroelectric properties achieved in this work essentially originates from more significant changes in crystal spontaneous polarization intensity with temperature. In other words, it results from increased primary pyroelectric effect, rather than factors such as introduced stress, changes in thermal conductivity, or capacitance.

Furthermore, LN-2# samples with significantly enhanced pyroelectric coefficients were heat annealing-treated in an air atmosphere at 373 K for 7 days (sample number: LN-2#-1), and were stored at room temperature for one year (sample number: LN-2#-2). The properties of LN crystals showed no significant changes (shown in Table 1). This also ensures that the detection performance of LN crystals with optimized pyroelectric properties will not degrade during extended operational periods in practical applications.

The application potential of pyroelectric materials is assessed quantitatively through figures of merit (FOMs) [29]. For voltage-mode pyroelectric materials, the voltage responsivity figure of merit ($F_{V}\;=\;p/C_{v}\varepsilon_{0}\varepsilon_{r}$) and the detectivity figure of merit ($F_{D}\;=\;p/C_{v}\sqrt{\varepsilon_{0}\varepsilon_{r}\tan\;\delta }$) are typically used to compare different materials. Table 2 presents the calculated FOMs for LN crystals at different reduction states, along with the impedance of sensing elements compared with PZT ceramic (Ref. [30]).

At room temperature, the F_V_ of LN crystals was improved by 1.8~3.2 times, while F_V_ was improved by 1.6~3.4 times. By optimizing the reduction annealing process, the pyroelectric coefficient was significantly enhanced while specific heat capacity, dielectric constant, and dielectric loss remained essentially unchanged. This resulted in substantially improved detection performance of optimized LN crystals. Among these, LN-1#, LN-2# and LN-3# all outperformed commercial PZT ceramic materials, achieving practical application. Additionally, LN-2#, and LN-3# exhibited impedance matching with the input impedance of the preamplifier while simultaneously achieving enhanced pyroelectric coefficients.

### 3.2. Pyroelectric Response of Lithium Niobate Crystals for Infrared Detection

Voltage responsivity $R_{V}\;={\;V}_{S}/P_{I}$ is commonly used to characterize the ability of a detector to convert infrared radiation signals into voltage signals. In this work, the temperature of the blackbody radiation source and its distance from the detector were fixed. Therefore, the power of the infrared radiation source $P_{I}$ remained constant. Differences in response voltage $V_{S}$ directly reflected differences in detector performance.

The PIRDs of LN crystals were prepared from RAW-LN, LN-1#, LN-2#, LN-3#, and LN-4# samples, the detector sample numbers are the same to the crystal sample numbers. The response voltages of each detector were measured at mechanical chopper modulation frequencies of 0.1 Hz, 1.0 Hz, and 3.0 Hz. All positive rising signals correspond to the infrared radiation input response, and negative falling signals correspond to the infrared radiation removal response. For comparison with commercial PZT pyroelectric detectors, the response voltage was measured for the commercial PIRDs of PZT ceramic detectors under this test system.

**Figure 4:**
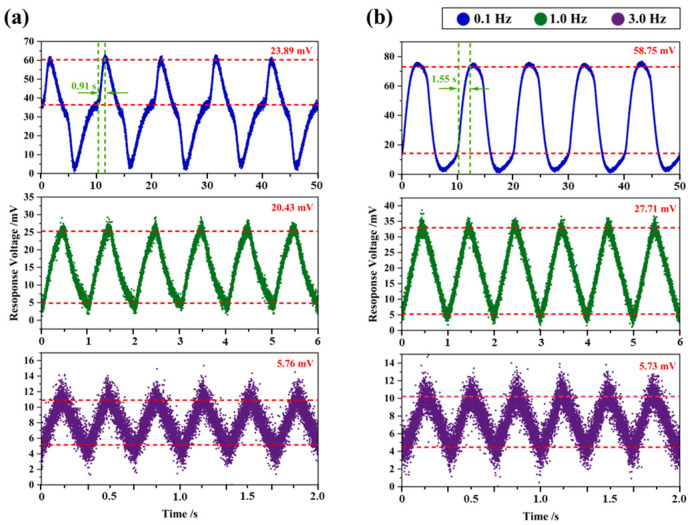
Response voltage of commercial PZT pyroelectric infrared detectors (D203S) at 0.1 Hz (blue), 1.0 Hz (green), and 3.0 Hz (purple), where (**a**) is PZT-1# and (**b**) is PZT-2#.

Two distinct response behaviors were observed in the test results of the PZT detectors, and the difference was particularly pronounced at a modulation frequency of 0.1 Hz, as shown in Figure 4. PZT-1# had a faster response rate at 0.1 Hz, reaching peak response in about 0.91 s, and its response voltage had already reached equilibrium before the infrared radiation changed. PZT-2# exhibited about 1.7 times longer than PZT-1# to reach the peak response, and its signal decayed more slowly after peaking, resulting in a response voltage at 0.1 Hz being approximately 2.5 times that of PZT-1#. At modulation frequencies of 1.0 Hz and 3.0 Hz, the response voltages of both detectors were basically the same, at approximately 20 mV and 5 mV, respectively.

The response rate of detectors to infrared radiation signals is primarily determined by the electrical time constant $\tau_{E}\;=\;\mathrm{RC}$. This variation stems from differences in the material intrinsic capacitance *C* of materials, and impedance mismatch with the preamplifier input impedance. The detection speed to reach thermal equilibrium with its environment is governed by the thermal time constant $\tau_{T}\;=\;M/G$, which correlates with the heat capacity *M* and thermal conductivity *G*. Therefore, the response behavior difference between PZT-1# and PZT-2# at 0.1 Hz may be attributed to the larger thickness of the PZT-2# sensing element. This structural difference affects the time required for the response voltage to return to equilibrium under extremely low-frequency conditions. As the operating frequency increases, the electrical response process gradually becomes dominant, and the response voltage depends primarily on the FOMs of materials.

**Figure 5:**
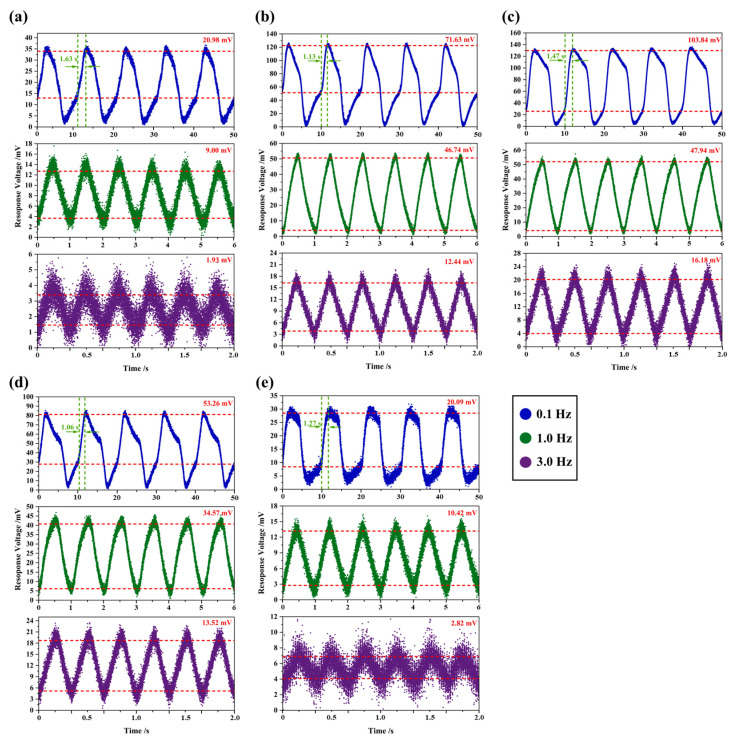
Response voltages of LN crystal pyroelectric infrared detectors at 0.1 Hz (blue), 1.0 Hz (green), and 3.0 Hz (purple), where detectors (**a**–**e**) are designated as RAW-LN, LN-1#, LN-2#, LN-3#, and LN-4#, respectively.

The test results for PIRDs based on LN crystals are shown in Figure 5. For the RAW-LN and LN-1# crystal samples, the output signal of the detectors was nearly zero due to an impedance mismatch. Therefore, a 100 GΩ chip resistor was packaged between the source and ground of the JFET to achieve impedance matching. All other detectors lacked match resistors.

The low pyroelectric coefficient of the RAW-LN sensing element resulted in a lower response voltage compared to the PZT detector, and its detection performance degraded with increasing modulation frequency. At a modulation frequency of 3.0 Hz, the response voltage was only 1.93 mV. The pyroelectric properties of the LN-4# sensing element were similar to RAW-LN; however, its reduced resistivity after reduction annealing enabled comparable detection performance to RAW-LN without requiring a matching resistor. The pyroelectric coefficient of the LN-1# sensing element was significantly enhanced, with its F_V_ nearly doubled that of PZT ceramic, the detector achieved response voltage 1.7 to 2.3 times that of PZT at 1.0 Hz, and approximately 2.2 times at 3.0 Hz. The LN-3# sensing element exhibited a slightly lower pyroelectric coefficient than LN-1#, but the detector required no external matching resistor, the response voltage was approximately 25% lower than that of LN-1# at 0.1 Hz and 1.0 Hz; while it matched LN-1# at 3.0 Hz, its performance was also superior to PZT detectors. The LN-2# sensing element showed a significantly enhanced pyroelectric coefficient, nearly tripled F_V_, and its response voltage was 1.7 times that of PZT-2# at 0.1 Hz and 1.0 Hz, the response rate was also faster, and at 3.0 Hz, the response voltage was 2.8 times that of PZT-2#.

In summary, PIRDs of LN crystals optimized for pyroelectric properties exhibited superior detection performance compared to commercial PZT detectors, achieving practical application standards. Additionally, based on the distribution characteristics of test data in scatter plots, these detectors showed low noise. According to the thermal time constant formula, reduced thickness decreases thermal inertia, thereby shortening the time for the sensing element to reach thermal equilibrium with the environment. This improves the performance of the detectors. If the sensing element thickness is further optimized and reduced to decrease its capacitance, the response rate could be improved.

## 4. Conclusions

This work performed defect modulation on LN crystals through a controllable reduction annealing treatment. By regulating the escape of oxygen atoms at different positions within the LN crystals, the pyroelectric properties were optimized, and the pyroelectric coefficient increased by more than three times. This performance was comparable to commercial PZT ceramic materials. Based on the significant improvement in the pyroelectric effect, the FOMs of LN crystals improved by more than three times at room temperature, representing approximately 2~4 times higher performance than commercial PZT ceramic materials. Analysis of thermal properties and electrical properties of LN crystals at different reduction annealing states, revealed that the enhancement of the primary pyroelectric effect is the fundamental factor in the improvement of FOMs in LN crystals. Performance degradation during long-term operation was also not observed. This result is also the optimal value reported publicly for LN crystals to date. Concurrently, synergistic control had been achieved to increase the pyroelectric coefficient while decreasing the resistivity of the LN crystal, enabling matching with the input impedance of detector preamplifiers. Pyroelectric infrared detectors prepared from optimized LN crystals need no external matching resistors. Operating across the 0.1 Hz to 3.0 Hz operating frequency range, these detectors achieved superior response voltages compared to commercial PZT detectors throughout the entire frequency band, featuring faster response rates and lower noise levels.

## Figures and Tables

**Table 1:** The properties of LN crystals after reduction annealing treatment.

Sample	*p* (10^−4^ C/m^2^K)	Thermal Properties			Electric Properties			
		*α_z_* (10^−6^ m^2^/s)	*k_z_* (W/mK)	*C_v_* (10^6^ J/m^3^K)	*ρ* (Ωm)	*ε_r_* @1 kHz	tan*δ* @1 kHz	*d*_33_ (10^−12^ C/N)
RAW-LN	1.066	1.577	4.179	2.650	3.89 × 10^13^	28.844	0.0027	12.7
LN-1#	2.261	1.544	4.088	2.648	1.50 × 10^13^	28.605	0.0030	12.9
LN-2#	3.362	1.573	4.197	2.668	3.08 × 10^12^	28.345	0.0023	13.1
LN-3#	1.943	1.507	4.198	2.786	8.68 × 10^11^	27.430	0.0030	12.9
LN-4#	1.034	1.501	4.056	2.702	3.60 × 10^11^	27.810	0.0033	13.1
LN-5#	0.895	1.495	4.094	2.738	1.11 × 10^11^	28.699	0.0023	12.8
LN-6#	0.737	1.511	4.165	2.756	8.38 × 10^10^	28.585	0.0022	12.8
LN-2#-1	3.373	1.528	4.187	2.740	4.05 × 10^12^	28.313	0.0021	12.9
LN-2#-2	3.366	1.553	4.174	2.688	4.44 × 10^12^	28.448	0.0022	13.0

**Table 2:** The figures of merit of LN crystals and impedance converted to the sensing elements.

Sample	F_V_ (m^2^/C)	F_D_ (10^−5^ Pa^−1/2^)	*Z* (Ω)
RAW-LN	0.048	4.845	1.04 × 10^16^
LN-1#	0.103	9.798	4.00 × 10^15^
LN-2#	0.154	16.590	8.22 × 10^14^
LN-3#	0.088	8.172	2.31 × 10^14^
LN-4#	0.048	4.246	9.59 × 10^13^
LN-5#	0.039	4.277	2.97 × 10^13^
LN-6#	0.032	3.585	2.24 × 10^13^
PZT	0.061	4.05	5.21 × 10^14^

## Data Availability

The data that support the findings of this work are available from the corresponding author upon reasonable request.
